# Trends in demographic and health survey publications based on a bibliometric analysis

**DOI:** 10.1080/16549716.2026.2680363

**Published:** 2026-06-01

**Authors:** Evans Omondi, Symon M. Kariuki, Soumaila Ouedraogo, Rachel Odhiambo, Daniel Osuka, Eliud Wekesa, Patricia Kitsao-Wekulo, Agnes Kiragga, Catherine Kyobutungi

**Affiliations:** a African Population and Health Research Center, Nairobi, Kenya; b Institute of Mathematical Sciences, Strathmore University Nairobi, Nairobi, Kenya; c Department of Sociology and Community Development, South Eastern Kenya University, Kitui, Kenya

**Keywords:** Demographic and Health Surveys (DHS), evidence generation, global health, publication delays, temporal trends

## Abstract

**Background:**

The Demographic and Health Surveys (DHS) Program, launched in 1984, provides high-quality population health data that underpins a vast body of global health research. However, the scale and growth patterns of DHS-based publications remain underexplored, particularly as donor funding uncertainties threaten program sustainability.

**Objective:**

We examine temporal trends in DHS-based research output from 1984 to 2025, quantifying growth patterns and publication delays to inform understanding of the program’s global research expansion.

**Methods:**

A systematic bibliometric review was conducted following PRISMA guidelines across PubMed, Scopus, Web of Science, Dimensions, Wiley, and CINAHL. Eligible peer-reviewed articles using DHS data between 1984 and 2025 were identified. Annual publication counts were analyzed, segmented regression identified growth inflection points, and timeliness was assessed by calculating lag between survey completion and publication.

**Results:**

Over 10,000 DHS-based publications were identified. Annual output rose from isolated studies in the 1980s to several hundred annually by the 2010s. Segmentation analysis revealed two rapid growth phases: a 56-publications/year increase from 2004–2012, and a 71-publications/year increase from 2012 to 2024. Despite this growth, median lag from survey completion to publication remained approximately 5 years, with only a modest recent improvement (Kendall’s τ =  −0.623, *p* < 0.001).

**Conclusion:**

DHS data have fueled exponential growth in global health research over four decades, confirming their vital role in evidence generation. However, persistent publication delays highlight the need to shorten the pathway from data collection to dissemination through strengthened research capacity in low- and middle-income countries. Sustained funding is essential to maintain this critical evidence source.

## Paper Context

- **Main findings:** Over 10,000 peer-reviewed studies using Demographic and Health Survey (DHS) data were identified between 1984 and 2025, with publication output increasing markedly after 2004 and accelerating further after 2012. Despite this rapid growth in research output, the median time from survey completion to publication remains about 4 years, although it has improved substantially over time.
- **Added knowledge:** This study provides the most comprehensive and up-to-date bibliometric analysis of DHS-based research, documenting four decades of publication growth, identifying key inflection points in output trends, and quantifying improvements in the timeliness of translating DHS data into scientific evidence.
- **Global health impact for policy and action:** The findings highlight the critical role of DHS data in generating global health evidence and underscore the need for sustained funding and strengthened research capacity, particularly in low- and middle-income countries to ensure timely translation of survey data into policy-relevant insights.

## Background

The generation of robust, comparable, and high-quality population health data is fundamental to tracking global health progress, informing evidence-based policies, and guiding effective public health interventions [1]. Since its inception in 1984, the Demographic and Health Surveys (DHS) Program has served as a critical source of this global evidence base. Initially launched as a USAID-funded project to conduct a limited number of surveys, the DHS program began with a primary focus on fertility and child survival. The first wave of surveys in the mid-to-late 1980s were conducted in a modest number of countries, providing foundational but critical data for these nations [2]. Over the subsequent four decades, the program underwent remarkable expansion, both in geographical scope and thematic depth. From its initial focus, the DHS rapidly grew to encompass over 400 surveys in over 90 countries across Africa, Asia, Latin America, and the Middle East. The number of surveys conducted has proliferated significantly, with many countries now having implemented multiple rounds of DHS, such as the DHS-I, DHS-II, amongst other. Along with interim standardized surveys, the DHS has created a rich repository of longitudinal, cross-nationally comparable data on health and demographic trends [3].

DHS data underpin a significant proportion of global health research, enabling cross-country comparisons, longitudinal analyses, and investigations into disparities and determinants of health [4–6]. The contribution of the DHS to shaping our understanding of global health trends over the past four decades is undeniable. The breadth of health priorities covered has also expanded far beyond its initial scope to include maternal and child health, nutrition, mortality, gender, HIV/AIDS, and malaria, among others. It is unclear whether the recent scientific output of the repertoire is consistent with this expansion in scope and topics. This accessibility has, in turn, fueled a vast and expanding body of scientific literature, for which the increasing trend and policy impact over the years should be examined. However, despite the central role of DHS data in research, a comprehensive quantitative analysis of the scientific publications it has generated remains underexplored. There was an increasing trend in publication outputs by 2012 [2], which is testament to how these standardized methodology, rigorous implementation, and commitment to making data freely accessible have made the DHS an indispensable resource for researchers, policymakers, and program implementers worldwide [7]. While numerous studies have utilized DHS data to answer specific research questions, the meta-level trends, such as the scale, growth patterns, and temporal evolution of this entire corpus of literature, are not well documented recently. Understanding these bibliometric trends is crucial for assessing the program’s broader scientific impact and return on investment, to inform solicitation of new funding support following recent defunding by USAID [8].

Furthermore, this temporal analysis is of urgent practical importance. The DHS program, primarily reliant on donor funding, currently faces financial uncertainties and constraints [7,9,10]. Documenting the output and value of the research it enables is a powerful tool for advocacy, thereby providing tangible evidence of the program’s contribution to global knowledge. This temporal progress in the DHS program can be mapped against the introduction of funding or global health initiatives. Additionally, the efficiency of translating raw data into actionable evidence driven by topic outputs is critical. Tracking of the timeliness of research publication following data collection in the completed surveys is a key metric of this efficiency, with direct implications for the relevance of findings for contemporary policymaking.

To address these gaps, we conducted a systematic review and bibliometric analysis of peer-reviewed publications derived from DHS data from 1984 to 2025. We aimed to (i) quantify the temporal growth and volume of DHS-based scientific output, (ii) identify key inflection points in publication trends alongside the program’s global expansion, and (iii) assess the timeliness of research by analysing the lag between survey completion and subsequent publication. The trends in DHS publications elucidated by this study highlights the monumental impact of the DHS program on global health evidence, while also identifying challenges, such as publication delays, that must be addressed to maximize its future utility.

## Materials and methods

### Study design and data sources

We conducted a systematic bibliometric review to identify all peer-reviewed journal articles that utilized data from the Demographic and Health Surveys (DHS) program. The review was designed and reported in accordance with the Preferred Reporting Items for Systematic Reviews and Meta-Analyses (PRISMA) guidelines [11]. To ensure maximum coverage and minimize database-specific biases, a comprehensive search was performed across six major electronic bibliographic databases: PubMed, Scopus, Web of Science Core Collection, Dimensions, Wiley Online Library, and Cumulative Index to Nursing and Allied Health Literature (CINAHL). The search strategy was designed to capture all relevant publications from the inception of the DHS program in 1984 through to June 2025. The study is reported in accordance with the Preliminary Guideline for Reporting Bibliometric Reviews of the Biomedical Literature (BIBLIO); the completed checklist is provided as Supplementary file 1 [12].

### Search strategy

The search strategy was developed iteratively by the research team in consultation with an internal institutional specialist in systematic reviews. The core search terms included: ‘Demographic and Health Survey*’, ‘DHS’, ‘MEASURE DHS’, and the names of specific survey types (for example, ‘AIS’, ‘MIS’, ‘SPA’) to ensure sensitivity. The search terms were developed through an iterative process. Authors first generated a list of potential synonyms for DHS and its associated survey instruments. These candidate terms were then cross-referenced with the MeSH (Medical Subject Headings) database to identify controlled vocabulary equivalents. Unlike earlier bibliometric reviews of DHS publications such as Fabic et al. [2], which used a narrower set of terms focused primarily on the survey programme name, our strategy was deliberately broader, incorporating specific sub-survey acronyms (e.g. AIS, MIS, SPA) to improve sensitivity and capture a more complete corpus of DHS-derived literature, particularly from databases that may not index older terminology consistently. The search syntax was adapted to the specific requirements of each database, utilizing a combination of title, abstract, and keyword fields, and applying appropriate Medical Subject Headings (MeSH) terms where available. Then search sensitivity was improved by application search techniques such as truncation, and field tag. The complete search strings for all six databases, including all Boolean operators, field tags, truncation symbols, and MeSH terms applied, are provided in full in Supplementary file 2. The strings were adapted from the core search terms to meet each database’s specific syntax requirements.

### Eligibility criteria and study selection

Articles were considered eligible for inclusion if they were (1) published in a peer-reviewed journal; (2) reported on original empirical research; and (3) utilized microdata from any standard DHS survey (e.g. Standard DHS, Interim DHS, Malaria Indicator Survey, AIDS Indicator Survey) as a primary data source. Articles leveraging on one data source were considered eligible. We excluded review articles, methodological papers that did not analyze original DHS data, editorials, commentaries, conference abstracts, books, and theses to maintain a focus on primary research output. The DHS reports that were not yet published in mainstream peer review journals were not considered for analysis. Also excluded were incomplete PDF files, e.g. those missing metadata, such as authors dates and titles because this information was needed for analysis. The study selection process was conducted in two phases. First, all identified records were imported into the Covidence systematic review software [13] for deduplication. As covidence is compatible with major reference manager formats including MS Excel and PubMed text, original files were uploaded into Covidence as generated from the search databases. Deduplication was conducted in two stages. First, automated duplicate detection was performed by Covidence upon import, which identified and removed the majority of duplicate records. Second, any residual duplicates not detected by Covidence were identified and removed manually during data extraction and analysis. Second, a pair of independent reviewers (selected from the authorship list) screened the titles and abstracts of all unique records against the eligibility criteria. The articles to be selected were voluminous and therefore split or shared among the authors to reduce the workload. The full text of any potentially relevant article was then retrieved and assessed independently by the same pair of reviewers for a final inclusion decision. The names of the reviewers were listed against the articles to be screened. Any discrepancies at either stage were resolved through weekly discussion or, if necessary, by a third reviewer selected from the author list until all articles were reviewed in accordance with the study protocol.

We applied a rigorous multi-stage screening protocol to determine which studies were suitable for inclusion in our bibliometric and geospatial analyses. First, we excluded studies that were incomplete, or lacked metadata relevant for this analysis, e.g. year, place, and author details or if DHS was not the source of the data. To ascertain if the DHS was the primary source of the data, and no related platforms such as health and demographic surveillance systems, the articles were shared across the authors for manual screening. To manage false positives introduced by the broad search terms, particularly records related to the US Department of Homeland Security (DHS) sharing the same acronym, all authors were allocated a portion of records for manual screening. Each article’s methods section was reviewed to confirm that the data source was the Demographic and Health Surveys program and not a related surveillance system. To increase screening efficiency, Covidence’s built-in features were used, including keyword highlighting to flag DHS-related terms in the title and abstract, and custom tags to categorise records as confirmed DHS, probable DHS, or non-DHS for review. Records confirmed as non-DHS were excluded at this stage. Second, we removed duplicated articles, initially via Covidence automated detection, followed by manual detection during screening and analysis. Finally, we removed all articles that did not utilize primary empirical data for empirical analysis, including systematic reviews and editorials.

### Ethical considerations

As this study relies on publicly available bibliometric data, no ethical approval was required. However, we ensured proper attribution of all sources and adhered to citation ethics in reporting findings.

### Data analysis

The analytical approach was designed to characterize the evolving landscape of DHS-based research through three primary lenses: volume, growth trends, and timeliness. First, a descriptive analysis was conducted in which the annual number of publications was tabulated. This simple count was plotted over time to visualize the overall growth trajectory from the program’s inception in 1984 through the end of our analysis period in 2025.

Second, we employed segmented regression analysis, also known as join-point regression [14] to systematically identify inflection points (join-points) in a temporal trend where the rate of increase or decrease in annual publication counts changes significantly. The model fits a series of connected linear segments to the data, allowing the quantification of the annual percentage change and absolute change in the number of publications for each distinct period and statistical testing of whether these changes in the trend were significant.

Third, a separate analysis was conducted to assess the timeliness of the research pipeline. For each article, a publication lag was calculated by subtracting the year of the survey’s completion from the year of the article’s publication. The recent survey date was used, to calculate publication time lags, where the published paper utilized multiple surveys, pooled datasets, or multiple countries/years. Survey year was obtained from the articles, and when not available the survey details were checked against the DHS repositories. The distribution of these lag times across the entire corpus of literature was summarized using medians and inter-quartile ranges (IQRs), which provide a robust measure of central tendency and spread resistant to outliers. The timeliness of publications was categorized into rapid (≤2 years), timely (3–5 years), delayed (6–10 years) and very delayed (>10 years), based on central tendency distributions, consensus discussions among the authors as well on empirical evidence on median time lags from the literature [15]. Trends in this publication lag over the decades were evaluated both visually, using scatter plots and smoothed trend lines, and statistically using non-parametric tests for trend to determine if data-to-publication delays have improved over time.

All the statistical computations and graphical outputs were generated using R (version 4.6.0, R Core Team) and R Studio (version 2026.04.0 + 526, R Studio Team) software.

## Results

### PRISMA search results

An extensive search strategy conducted across several academic databases identified 53,644 publications related to Demographic and Health Survey (DHS) research. Web of Science identified most of the articles (*N* = 14,222 (26.5%)), followed by PubMed (*N* = 13,108 (24.4%), with Dimensions producing the least (*N* = 500 (0.9%)). In the initial screening step, 11,845 duplicate entries were removed.

The deduplicated records were merged and then assessed through a careful review of titles and abstracts. During this process, 30,431 publications were excluded because they appeared in more than one database or lacked essential metadata, such as complete author affiliations or citation information, which resulted in exclusion of a further 1238 articles.

**Figure 1.**
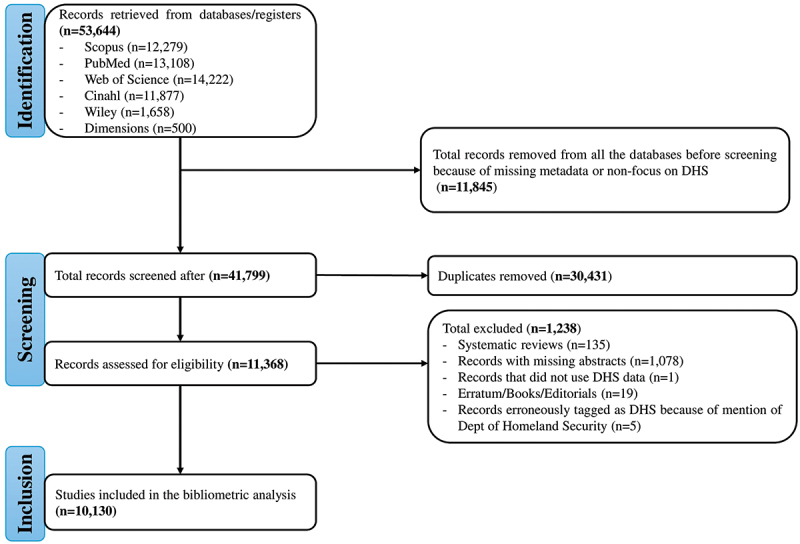
PRISMA flow diagram illustrating the identification, screening, and inclusion process. Records were retrieved from six databases (Scopus, PubMed, web of Science, CINAHL, Wiley, Dimensions; total *n* = 53,644). Following removal of records with missing metadata or no focus on DHS (*n* = 11,845) and deduplication (*n* = 30,431), a total of 41,799 records were screened. After title and abstract screening, 11,368 records were assessed for full-text eligibility; 1,238 were excluded for the reasons shown. A total of 10,130 studies were included in the final bibliometric analysis.

Ultimately, 10,130 studies satisfied all inclusion requirements and were retained for analysis. The full identification, screening, and inclusion pathway are outlined in Figure 1, which adheres to the PRISMA reporting framework.

### Temporal trends in DHS-Based publications

**Figure 2.**
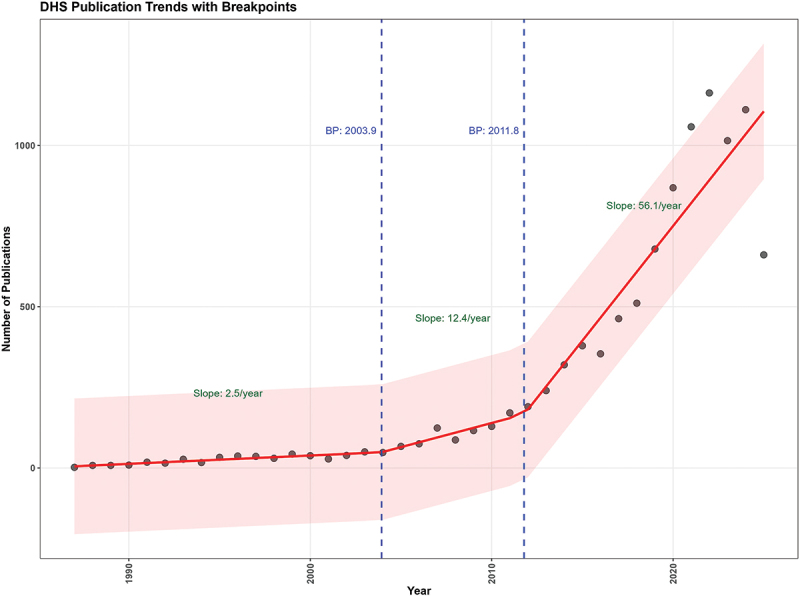
Annual DHS-based publication counts from 1984 to 2025, with segmented regression trend lines and identified breakpoints. Each point represents the total number of peer-reviewed publications using DHS data in that year. The red line shows the fitted segmented regression model (R^2^ = 0.92). Dashed vertical lines mark the two statistically identified breakpoints at approximately 2004 and 2012, which divide the series into three distinct growth phases. The shaded band represents the 95% confidence interval around the fitted trend. Slopes for each phase are annotated.

A segmented regression analysis was performed to identify significant inflection points and changes in the temporal trend of DHS publication output. The model identified two statistically significant breakpoints, partitioning the time series into three distinct phases as shown in Figure 2.

The model exhibited an excellent fit to the data, explaining 92% of the variance in publication counts (*R*^2^ = 0.92, adjusted *R*^2^ = 0.908). The estimated breakpoints, representing years where the publication trend changed significantly, occurred in 2003.93 (approximately 2004) and 2011.78 (approximately 2012). The model parameters are presented in Table 1.

**Table 1.** Segmented regression results for temporal trends in DHS-based publications. The model coefficients (base slope, U1.PubYear, U2.PubYear) represent changes in slope relative to the preceding period, not the absolute rate of growth within that period. The resulting slopes for each phase are as follows: phase 1 (pre-2004): 2.6 publications/year (Base slope); phase 2 (2004–2012): 15.0 publications/year (Base slope + U1 = 2.6 + 12.4); phase 3 (2012–2024): 71.1 publications/year (Base slope + U1 + U2 = 2.6 + 12.4 + 56.1). Only the phase 3 slope change was statistically significant (*p* = 0.004).

Model Components	Estimate	SE	95% CI	t value	Pr(>|t|)
First Breakpoint (Year)	2003.9	7.2	[1989.9, 2018.0]	–	–
Second Breakpoint (Year)	2012.8	1.7	[2008.4, 2015.2]	–	–
Intercept	−5103.5	10,596.9	[−25873.5, 15,666.5]	−0.5	0.633
Base Slope (PubYear)	2.6	5.3	[−7.8, 13.0]	0.5	0.632
First Slope Change (U1. PubYear)	12.4	17.4	[−21.7, 46.5]	0.7	0.481
Second Slope Change (U2. PubYear)	56.1	18.0	[20.8, 91.5]	18.0	0.004

R2 = 0.92, Adj. R2 = 0.91, Residual SE = 107.3.

The base slope of 2.6 publications per year was not statistically significant (*p* = 0.632). This suggests that before the first break point in 2004, there was no strong evidence of a consistent upward or downward trend in the annual number of DHS publications. The publication output during this era was relatively stable. Following the first breakpoint in 2004, the model estimated a positive change in slope of +12.4 publications per year (95% CI: −21.7 to 46.5; *p* = 0.481). This change was not statistically significant at conventional levels. However, the combined slope for this period, representing the total rate of change (Base Slope + U1 = 15.0 publications/year), suggests a modest acceleration in output. Given the wide standard error for this coefficient (SE = 17.4), the evidence for a distinct growth phase between 2004 and 2012 should be interpreted with caution. This period likely reflects the expanding scope and adoption of the DHS program, increased funding, and its establishment as a reliable data source to monitor health and demographic indicators in low- and middle-income countries [7]. The most dramatic and statistically significant shift occurred at the second break point in 2012. The slope change for this phase was +56.1 publications per year (*p* = 0.004). This resulted in a total slope for the final period of approximately 71.10 publications per year (Base + U1 + U2). This represents a hyper-growth phase where the annual publication output increased exponentially. To aid interpretation, the resulting absolute growth rate for each phase, combining the base slope with successive slope-change coefficients, was 2.6 publications/year before 2004, 15.0 publications/year between 2004 and 2012 and 71.1 publications/year between 2012 and 2024.

### Publication and survey timeliness analysis

**Figure 3.**
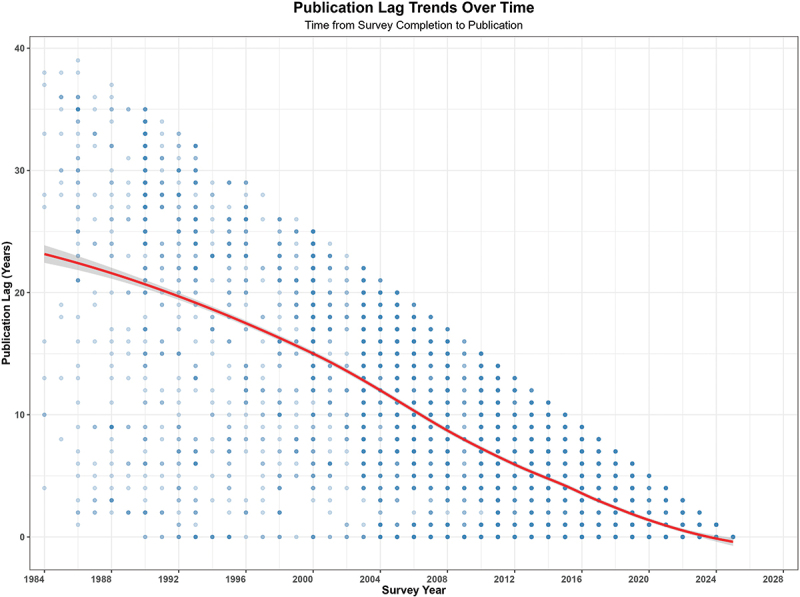
Scatter plot showing the time lag (in years) between DHS survey completion and peer-reviewed publication, plotted against the survey year. Each point represents one article. The red smoothed line shows the overall trend with a 95% confidence band. A downward trajectory indicates that more recent surveys are being published with shorter delays. Points clustered near zero in the 2020s reflect either genuinely faster dissemination or incomplete follow-up time for the most recent surveys (right truncation).

Figure 3 displays the relationship between the survey year and the time lag from the survey completion to publication. Each point represents the lag of an individual survey, and the red smoothed line shows the overall trend with a confidence band of 95%. Publication lag was longest for surveys conducted in the mid-1980s, with many taking more than 30 years to reach publication. The lag decreases steadily for later survey years, falling to roughly 10 years for surveys around 2010 and continuing to decline thereafter. By the early 2020s, the median lag approaches zero, indicating that analyses of recent surveys are being published within a few years of data collection, which may be because these datasets have not been fully exploited or followed up for analysis and publication. The continuous downward trajectory of the smoothed curve highlights a consistent acceleration in the translation of survey data into peer-reviewed outputs over the four decades represented.

**Figure 4.**
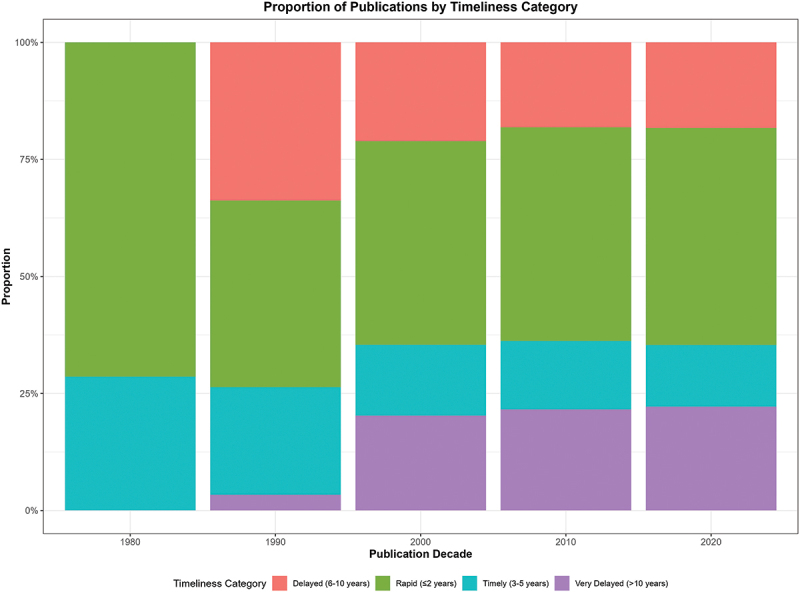
Stacked bar chart showing the proportion of DHS-based publications falling into four timeliness categories by decade of publication. Categories are defined as: rapid (≤2 years from survey completion to publication); timely (3–5 years); delayed (6–10 years); and very delayed (>10 years). Category boundaries were determined based on central tendency distributions in the data and empirical evidence on median publication time lags from the literature [15]. Colours correspond to each category as shown in the legend.

The stacked bar chart presented in Figure 4 shows the proportion of publications in four timeliness categories across publication decades. In the 1980s, nearly all studies were either rapid (≤2 years) or timely (3–5 years). From the 1990s onward, the share of delayed publications (6–10 years) and very delayed publications (>10 years) rises sharply and remains substantial through the 2020s. By the most recent decade, roughly three quarters of studies fall into the rapid or timely groups, but a persistent minority continue to experience delays exceeding a decade.

**Figure 5.**
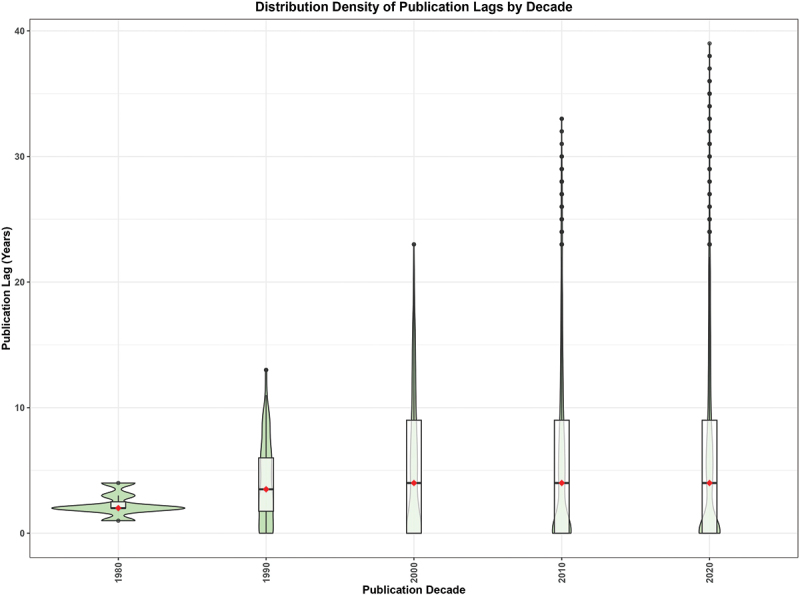
Violin plots showing the distribution density of publication lags (in years from survey completion to publication) by decade of publication. The width of each violin reflects the density of observations at each lag value. The box within each violin represents the interquartile range; the red dot indicates the median lag. Extended upper whiskers in the 2000s, 2010s, and 2020s reflect a subset of publications with very long delays, likely representing re-analyses of older surveys.

Figure 5 illustrates the distribution of publication lags by decade of publication. Median lag times (red dots) increase steadily from the 1980s through the 2000s and then stabilize, with wide inter-quartile ranges and numerous long upper whiskers in the 2000s, 2010s, and 2020s. While a small subset of studies in later decades show very short lags, the elongated upper tails indicate that many publications continue to experience extended delays exceeding 20 years.

The findings in Table 2 reveal a strong and statistically significant negative temporal trend in publication lag, as evidenced by Kendall’s τ of −0.623 (*p* < 0.001). The median lag between survey completion and research publication has decreased substantially over the decades, from 23 years in the 1980s to 21 years in the 1990s, 13 years in the 2000s, and 4 years in the 2010s. This trend has culminated in a median lag of 0 years for publications in the 2020s, indicating a dramatic temporal improvement in the timeliness of research dissemination. The overall median lag across all decades is 4.0 years, with an inter-quartile range of 9.0 years.

**Table 2.** Time from DHS survey completion to research publication.

Period	Surveys	Median Lag	IQR
Overall	9007	4.0	9.0
1980s	168	23.0	22.2
1990s	690	21.0	16.0
2000s	1734	13.0	10.0
2010s	3916	4.0	7.0
2020s	2499	0.0	0.0

Kendall’s τ = −0.623, p < 0.001.

## Discussion

The findings from this analysis show that publication outputs from the DHS have significantly increased as its inception in 1984, although this was not remarkable until 2004, when a 56 publications/year increment was documented, and again in 2012, when the increase was substantially greater at 71 publication/year. Prior to the first break point in 2004, there was no strong evidence for a consistent upward or downward trend in the annual number of DHS publications. This is consistent with the highest median lag or delays in publication, of 23 years and 21 years for DHS surveys, completed in the 1980s and 1990s. Access to scientific mentorship, capacity building in scientific writing, and publication resources likely influenced the growth in publication output [16].

The change periods in publications likely represent three distinct evolutions in the advancement of global health. The formative stages of 1984–2004 represent a growth phase when the focus was solicitation of funding and on-boarding of countries into the DHS program. The publication output during this era was relatively stable or constant because there was probably more emphasis and focus on strengthening of scientific infrastructure and capacity. For example, this is when (1996) the Good Clinical Practice, which has become universally useful for ethical conduct of public health research was published [17]. The growth phase of between 2004 and 2012 is when the DHS program not only on-boarded more countries, following increasing funding, but also incorporated more health conditions. The global health advances including interests in nutritional health in 2006, for example, may have the DHS program [18]. There were also concerted efforts for alternative options for raising resources for health during this period, which may have translated to more research and publications [19]. The expansion phase from 2012 to 2025, happened when focus was extended to emerging issues health and well-being priorities. Many global health initiatives that could shape the focus of the DHS program were introduced during this growth phase, including elimination of mother-to-child transmission of HIV and syphilis and sustainable development goals in 2015 [20,21]. It is worth noting that global health milestones that drove publications in each growth period may have been initiated earlier but had lasting implications in the periods thereafter, for example the Global Fund, which was launched in 2001 and whose prevention, treatment and care programs are still impactful and useful to date [22].

There are other factors that can influence DHS scientific outputs at any points of the period of growth. Number of publications over the years may also be driven by the number of surveys carried out over the period, which can be confirmed through focused in-country analysis. We found that number of surveys appeared to increase over the analysis period. This notable increase may be related to change in scope of DHS over the period that may have contributed to the elaborate growth in DHS scientific output. While the focus was on fertility family planning issues at the inception of the DHS program, in the 1990s, there was the addition of new modules of domestic violence and malaria, with capacities to test both infectious, e.g. measures and non-communicable diseases, e.g. diabetes [2]. This module introduction corresponded with the WHO advocacy for the need to shift from infectious to non-communicable diseases [23]. Significant increase in publication outputs was probably because of the addition of core questionnaires for these maternal child health and HIV/AIDS after 2000. Coincidentally, around this period in 2001, the UN Declaration of Commitment on HIV/AIDS was made [24]. Funder interest in an area DHS focus, and priorities of a country hosting the DHS program may drive the DHS outputs.

The highlighted growth in DHS publications may in part be related to significant increase in funding. For example, evidence in the literature shows that the spending of USAID, the main funder of the DHS, increased by 68% per country, and by 97% per capita over the analysis period [8]. This funding investment was sustained during this entire period of growth and expansion in the DHS, when the observed publication growth was measured. Other wealthy countries, e.g. United Kingdom, and the Netherlands also made funding commitments toward DHS program, when other global health investment initiatives were on the increase especially after 2001 [25], that targeted health challenges similar to those of the DHS program, e.g. malaria, and women and children-related health-related issues. Because of this sustained funding, for example, DHS enrollment has grown from a handful of countries at its inception to 68 in 2002, and to over 90 countries presently in the program. This increase in funding has accelerated the adoption of the DHS program by many LMICs, especially in the first phase of the publication growth, leading to its establishment as a reliable data source for monitoring health and demographic indicators in low- and middle-income countries [7].

The publication lag observed can be interpreted in several ways, dependent on the time since the completion of surveys, and the periods of publications. Time since the survey showed positive correlation with the publication time lag, for example, for surveys completed in the 1980s, it would take as many as 30 years to get some articles published. It would follow therefore that the continuous downward trajectory of the smoothed curve highlights a consistent acceleration in the translation of survey data into peer-reviewed outputs over the four decades represented. This increase is consistent with scientific outputs reported for public health journals overall in the past 30 years [26]. Even if the publication lag periods for the later years are small, likely due to prompt publication since completion of recent surveys, this finding may also indicate that limited DHS datasets have been translated into manuscripts for publications or that there has been fewer follow-up surveys or studies. The extended uncertainty plots (long-tailed whiskers) for the publication lags in recent years suggest that a few unpublished manuscripts were carried over from earlier years. Many publications in the 1980s and 1990s were under the rapid or timely publication category, implying there were few outputs back then and that the few that delayed accumulate over long periods. This delayed publication or carryover publications from previous years, calls for the need to build scientific capacity especially in LMICs, to ensure the problem is not extended into the future. While the DHS invested heavily in capacity building most of it was related to operational aspects of the study e.g. investment in survey and data capture software, and methodological aspects of sampling and tool adaptation and administration, overlooked individual capacity building and capacity strengthening for data science and scientific writing, particularly in LMICs.

## Strengths and limitations

This study provides the most up-to-date assessment of trends in DHS scientific outputs, offering the first bibliometric analysis to extend through 2025 and the first conducted in the context of the program’s recent defunding. By employing a comprehensive multi-database search strategy going beyond the single database approaches used in earlier reviews such as Fabic et al. [2] and applying segmented regression alongside publication timeliness analyses, this review presents a more complete and contemporary understanding of the DHS program’s scientific contribution. Unlike previous syntheses, it captures recent publication surges, and changes in publication lag, while situating these trends within evolving global health funding landscapes. Together, these advances provide timely evidence on the sustained relevance and growing influence of DHS data and offer useful metrics for assessing the impact of major health initiatives introduced during key growth periods. However, this review did not incorporate grey literature, which may disproportionately exclude outputs from LMIC scholars and institutions. Furthermore, the analysis relies on the accuracy and completeness of database indexing, meaning that inconsistencies in metadata, keyword mapping, or journal coverage could have influenced the comprehensiveness of the publication corpus.

## Conclusion

The findings show scientific expansion in the use of DHS data and publications, although disparities still exist in growth and timeliness of publication, which can be addressed through capacity building in scientific leadership. The impact of these DHS publications on decision-making by policymakers and local scientific leadership of publications should be evaluated in a separate report. If funding is available, the priority health conditions in the DHS program should also be expanded to accommodate important emerging problems such as mental health and health implications of the climate change [27]. The findings underscore the necessity of sustained and diversified funding to ensure the continuity of the vital DHS program and to support the ongoing availability of critical data for global health advancement. Availability of funding is crucial in supporting systems and capacity building for maintaining data quality and writing dissemination reports, especially in LMIC. The efforts to obtain funding to sustain DHS program will involve many wider actors, especially the funders, recipient countries, advocates of global health initiatives and influential NGOs.

## Supplementary Material

Supplementary_file_2.docx

Supplementary_file_1.docx
