# Supplementary material for: Trends in demographic and health survey publications based on a bibliometric analysis
Source: Glob Health Action. 2026 Jun 1;19(1):2680363. doi: 10.1080/16549716.2026.2680363 (PMC13228172; doi:10.1080/16549716.2026.2680363)
Supplement: Supplementary_file_2.docx [file ZGHA_A_2680363_SM9629.docx]

**Trends in Demographic and Health Survey Publications Based on a Bibliometric Analysis**

**Supplementary File 1: Full Database Search Strategies**

*Ms. No. 261503966 | Global Health Action*

**Overview**

This file presents the complete search strings used across all six bibliographic databases searched for this bibliometric analysis. Searches were conducted between May and June 2025, covering publications from the inception of the Demographic and Health Surveys (DHS) program in 1984 through to June 2025. The search strategy was developed iteratively: authors first generated a list of synonyms and related terms, which were then cross-referenced with the MeSH (Medical Subject Headings) database to identify controlled vocabulary equivalents. Search syntax was adapted to the specific field tags and Boolean requirements of each database. Sensitivity was enhanced through truncation and the inclusion of specific DHS sub-survey acronyms (AIS, MIS, SPA).

**The core search terms used across all databases were:**

• "Demographic and Health Surveys" (full name)

• "Demographic Health Survey*" (truncated variant)

• "MEASURE DHS" (program name used in earlier iterations)

• "DHS" (abbreviation - see false positive handling note below)

• "AIDS Indicator Survey" / "AIS"

• "Malaria Indicator Survey" / "MIS"

• "Service Provision Assessment" / "SPA"

• Demograph* AND "Health Survey*" (broad combinatorial term)

| **False Positive Handling: Exclusion of 'Department of Homeland Security' Records**  Because the abbreviation 'DHS' is shared by the Demographic and Health Surveys program and the US Department of Homeland Security, searches using 'DHS' as a standalone term returned a number of irrelevant records. To manage this systematically, all co-authors were allocated a portion of the deduplicated records for manual screening. Each author reviewed the methods section of each assigned article to confirm that the data source was a standard DHS program survey and not a related surveillance system or government agency. Covidence's keyword highlighting feature was used to flag the term 'DHS' in titles and abstracts, and custom tags were applied to categorise records as confirmed DHS, probable DHS, or non-DHS for team review. Records confirmed as non-DHS - including five records that referred exclusively to the Department of Homeland Security - were excluded at this stage, as noted in the PRISMA diagram (Figure 1 of the main manuscript). |
| --- |

**Detailed Search Strings by Database**

| **Database** | **Records retrieved (n)** | **Date searched** | **Fields searched** | **Full search string** |
| --- | --- | --- | --- | --- |
| **PubMed** | 13,108 | June 2025 | *Title, Abstract, MeSH Terms* | (  ("Demographic and Health Surveys"[MeSH Terms])  OR ("Demographic and Health Surveys"[All Fields])  OR ("Demographic Health Survey*"[All Fields])  OR ("DHS"[All Fields])  OR ("MEASURE DHS"[All Fields])  OR ("AIS"[All Fields] AND "health survey"[All Fields])  OR ("MIS"[All Fields] AND "health survey"[All Fields])  OR ("SPA"[All Fields] AND "health survey"[All Fields])  OR (Demograph*[All Fields] AND "Health Survey*"[All Fields])  )  *MeSH heading: "Demographic and Health Surveys" applied where available. Truncation (*) applied to "Demograph" and "Survey" to capture variant forms. Sub-survey acronyms (AIS = AIDS Indicator Survey; MIS = Malaria Indicator Survey; SPA = Service Provision Assessment) paired with "health survey" to reduce false positives. "DHS" paired contextually during manual screening to exclude records referring to the US Department of Homeland Security (see False Positive Handling below).* |
| **Scopus** | 12,279 | June 2025 | *Title, Abstract, Keywords (TITLE-ABS-KEY)* | TITLE-ABS-KEY (  "Demographic and Health Surveys"  OR "Demographic Health Survey*"  OR "MEASURE DHS"  OR "DHS" AND "health survey*"  OR "AIDS Indicator Survey" OR "AIS"  OR "Malaria Indicator Survey" OR "MIS"  OR "Service Provision Assessment" OR "SPA"  OR (Demograph* AND "Health Survey*")  )  AND PUBYEAR > 1983  *Boolean field tag TITLE-ABS-KEY() applied across title, abstract, and keywords. Truncation (*) used on "Survey" and "Demograph". Year filter restricts results to 1984 onwards, consistent with DHS programme inception.* |
| **Web of Science Core Collection** | 14,222 | June 2025 | *Topic (Title, Abstract, Author Keywords, Keywords Plus) — TS=* | TS=(  "Demographic and Health Surveys"  OR "Demographic Health Survey*"  OR "MEASURE DHS"  OR ("DHS" AND "health survey*")  OR "AIDS Indicator Survey" OR "AIS"  OR "Malaria Indicator Survey" OR "MIS"  OR "Service Provision Assessment" OR "SPA"  OR (Demograph* AND "Health Survey*")  )  AND PY=(1984-2025)  *Field tag TS= searches topic fields (title, abstract, author keywords, Keywords Plus). Publication year (PY) filter applied. Note: the original supplementary file incorrectly used S= instead of the correct TS= topic field tag; this has been corrected.* |
| **Dimensions** | 500 | June 2025 | *Title and Abstract* | Abstract and Title: (  "Demographic and Health Surveys"  OR "Demographic Health Survey*"  OR "MEASURE DHS"  OR "DHS"  OR "AIDS Indicator Survey" OR "AIS"  OR "Malaria Indicator Survey" OR "MIS"  OR "Service Provision Assessment" OR "SPA"  OR (Demograph* AND "Health Survey*")  )  *Dimensions does not support MeSH terms. Broad title and abstract search applied. The low yield (n=500) relative to other databases reflects Dimensions' indexing coverage of the specific journal corpus relevant to this search.* |
| **CINAHL (via EBSCOhost)** | 11,877 | June 2025 | *MeSH Subject Heading (MH), Title (TI), Abstract (AB), All Text (TX)* | (MH "Demographic and Health Surveys")  OR (TI "Demographic and Health Surveys" OR AB "Demographic and Health Surveys")  OR (TX "Demographic Health Survey*")  OR (TX "MEASURE DHS")  OR (TX "DHS" AND TX "health survey*")  OR (TX "AIDS Indicator Survey" OR TX "AIS")  OR (TX "Malaria Indicator Survey" OR TX "MIS")  OR (TX "Service Provision Assessment" OR TX "SPA")  OR ((TX Demograph*) AND (TX "Health Survey*"))  *CINAHL MeSH Subject Heading (MH) applied where the controlled vocabulary term exists. TX (All Text) field tag used for broader coverage of non-indexed terms. Truncation (*) applied to "Survey" and "Demograph".* |
| **Wiley Online Library** | 1,658 | June 2025 | *Title, Abstract, Keywords (Advanced Search)* | "Demographic and Health Surveys"  OR "Demographic Health Survey*"  OR "MEASURE DHS"  OR "DHS"  OR "AIDS Indicator Survey"  OR "Malaria Indicator Survey"  OR "Service Provision Assessment"  OR (Demograph* AND "Health Survey*")  *Wiley Online Library advanced search applied to Title, Abstract, and Keywords fields. Wiley does not support MeSH terms. Boolean operators applied within the platform's search interface.* |

**Summary of Records Retrieved**

| **Database** | **Records retrieved** | **% of total retrieved** | **Included in final analysis** |
| --- | --- | --- | --- |
| Scopus | 12,279 | 22.9% | — |
| PubMed | 13,108 | 24.4% | — |
| Web of Science Core Collection | 14,222 | 26.5% | — |
| CINAHL | 11,877 | 22.1% | — |
| Wiley Online Library | 1,658 | 3.1% | — |
| Dimensions | 500 | 0.9% | — |
| **Total (before deduplication)** | **53,644** | **100%** | **—** |
| **After deduplication and metadata exclusion** | **41,799** | — | — |
| **Final included studies** | **10,130** | — | **10,130** |

**Notes on Search Term Development**

**Term selection:** Search terms were developed iteratively. Authors first generated a list of potential synonyms for the DHS program and its associated survey instruments. These were cross-referenced with the MeSH database to identify controlled vocabulary equivalents. The final list was broader than that used in earlier reviews (e.g., Fabic et al. [2012]), deliberately incorporating specific sub-survey acronyms (AIS, MIS, SPA) to improve sensitivity and capture DHS-derived literature not always indexed under the full program name.

**Truncation:** The wildcard symbol (*) was used to capture variant word endings (e.g., 'Demographic*' captures 'Demographic', 'Demographics'; 'Survey*' captures 'Survey', 'Surveys', 'Surveyed').

**MeSH terms:** MeSH controlled vocabulary was applied in PubMed (MeSH Terms field) and CINAHL (MH field) where the heading 'Demographic and Health Surveys' exists. MeSH terms were not available in Scopus, Web of Science, Wiley, or Dimensions.

**Date limits:** All searches were limited to publications from 1984 (DHS program inception) through June 2025. No language restrictions were applied at the search stage; language was not used as an exclusion criterion.

**Field tags:** Each database uses different syntax for field-specific searching. Tags applied were PubMed [All Fields], [MeSH Terms]; Scopus TITLE-ABS-KEY(); Web of Science TS= (Topic); CINAHL MH, TI, AB, TX; Dimensions 'Abstract and Title'; Wiley advanced search Title/Abstract/Keywords.
